# Characterization of the complete mitochondrial genome of *Epipedocera atra* Pic (Cerambycidae: Cerambycinae: Tillomorphini)

**DOI:** 10.1080/23802359.2020.1844089

**Published:** 2021-01-05

**Authors:** Zishu Dong, Xiaolong Yi, Shengbo Li, Yueshi Li, Daxing Hu, Xialin Zheng, Wen Lu

**Affiliations:** a Guangxi Key Laboratory of Agric-Environment and Agric-Products Safety, National Demonstration Center for Experimental Plant Science Education, College of Agriculture, Guangxi University, Nanning, Guangxi, China; b Guangxi Wuzhou Rural Investment Development Co., Ltd, Wuzhou, Guangxi, China; c Xixian Senior High School Affiliated To Central China Normal University, Xinyang, Henan, China

**Keywords:** *Epipedocera atra*, mitochondrial genome, phylogenetic tree

## Abstract

*Epipedocera atra* is a common species of *Epipedocera* Chevrolat which distributed in South China and some countries in Southeast Asia. The complete mitochondria genome of *E. atra* was 15,662 bp in length, with 37 genes, including 13 protein-coding genes (PCGs), 22 tRNA genes (tRNAs), and 2 rRNA genes (rRNAs). The nucleotide composition was highly A + T biased, accounting for 70.34% of the whole mitogenome. Phylogenetic analysis indicated that *E. atra* had a close relationship with *Xylotrechus grayii* White.

## Introduction

*Epipedocera atra* is a common species of *Epipedocera* Chevrolat found in some parts of Southeast Asia and South China (Jiang et al. 1985; Hua et al. 2009). In this study, specimens of *E. atra* were collected from the Wenjing Village (22°87′N, 109°24′E) of Nanning City (Guangxi Autonomous Region, China) on a corn in middle florescence. The voucher samples were kept at the Guangxi Key Laboratory of Agric-Environment and Agric-Products Safety (Nanning, China), College of Agriculture, Guangxi University, Guangxi, China.

The total genomic DNA was extracted following the modified CTAB DNA extraction protocol and stored at Guangxi Key Laboratory of Agric-Environment and Agric-Products Safety (Nanning, China) with a sample number of SZHT0606G148. Then, a library was constructed and pair-end was sequenced (2*150 bp) with HiSeq (Illumina, San Diego, CA). Approximately 10.40 G of raw data and 10.30 G of clean data were obtained for sequence assembly by SPAdes (Version 3.9) with ‘-k 21,33,55,77 – careful’ option (Bankevich et al. 2012). The candidate was obtained by blast alignment of mitochondrial fragments from near-source species. By analyzing the positions of these candidate sequences in fastg graph and combining the adjacency sequence of these sequences in the graph, the complete loop path is obtained. The complete mitochondrial genome was obtained by extracting the sequence from the loop path diagram.

The complete mitochondrial genome of *E. atra* (GenBank accession number MT740323) revealed the size of 15,662 bp, containing A 38.41%, G 11.49%, T 31.93%, and C 18.17%. It is highly A + T biased, accounting for 70.34%, showing strong AT skew. The *E. atra* mitogenome contains 13 protein-coding genes (PCGs), 22 tRNAs (tRNA-Leu, tRNA-Gln, tRNA-Met, tRNA-Trp, tRNA-Cys, tRNA-Tyr, tRNA-Second Leu, tRNA-Lys, tRNA-Asp, tRNA-Gly, tRNA-Ala, tRNA-Arg, tRNA-Asn, tRNA-Ser, tRNA-Glu, tRNA-Phe, tRNA-His, tRNA-Thr, tRNA-Pro, tRNA-Second Ser, tRNA-Leu, tRNA-Val), and 2 rRNA (16S rRNA and 12S rRNA). The nucleotide sequence of 13 PCGs of all mitochondrial genes was 11,040 bp in length. The sizes of 2 rRNA genes were1049 bp and 775 bp, respectively. All of the 22 tRNAs, ranging from 62 to71 bp, have a classical cloverleaf structure.

Among these PCGs, 4 genes (*nad1*, *nad2*, *nad4l*, *atp8*) were with start codon ATT, 4 genes (*nad4*, *atp6*, *cox1*, *cox3*) have ATG as start codon, 2 genes (*cob, cox2*) have ATC as start codon, and 2 genes (*nad3*, *nad6*) were with start codon ATA. Besides, 8 PCGs (*nd2*, *nd4*, *nd4l*, *atp6*, *cox1*, *cox2* and *cox3*) used the stop codon TAA, and the remaining 5 genes (*nad1*, *nad3*, *nad6, atp8* and *cob*) were with the stop codon TAG.

**Figure 1.**
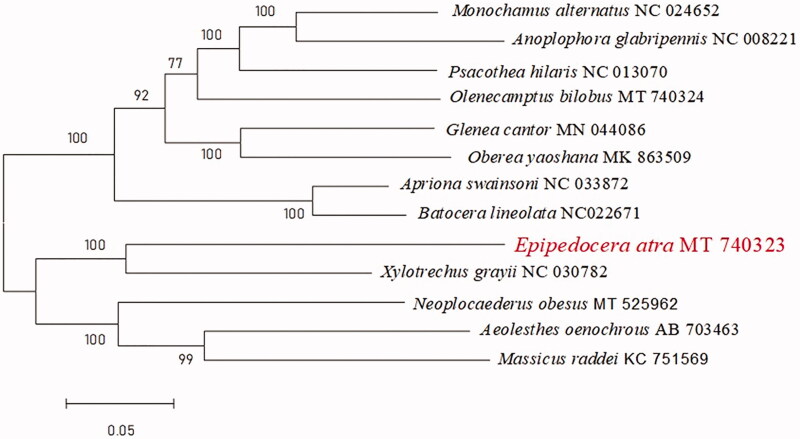
Maximum-likelihood phylogenetic tree of *E. atra* and other 12 species in Cerambycidae.

Molecular Evolutionary Genetics Analysis Version 7.0 (MEGA 7.0) was used to make phylogenetic inference among some Cerambycidae species by the maximum-likelihood method with 1000 bootstrap replicates (Kumar et al. 2016). Results show that *E. atra* is sister to *Xylotrexhus Chevrolat* (Figure 1).

## Data Availability

Mitogenome data supporting this study are openly available in GenBank at: https://www.ncbi.nlm.nih.gov/nuccore/MT740323. Associated BioProject, SRA, and BioSample accession numbers are https://www.ncbi.nlm.nih.gov/bioproject/PRJNA663275, https://www.ncbi.nlm.nih.gov/sra/SRX9145190, and SAMN16125319, respectively.
